# Pioneering Comprehensive Eye Care in Northern Pakistan: Development and Implementation of a Hub-and-Spoke Model

**DOI:** 10.7759/cureus.115674

**Published:** 2026-09-02

**Authors:** Zubaida Sirang, Raisa Khan, Shamila Ladak, Irfan Jeeva

**Affiliations:** 1 Ophthalmology, University of Calgary, Calgary, CAN; 2 Public Health Services, Aga Khan Health Service Pakistan, Karachi, PAK; 3 Medicine, Aga Khan University, Karachi, PAK; 4 Ophthalmology, Mid Yorkshire Teaching NHS Trust, Wakefield, GBR

**Keywords:** comprehensive eye care, hub-and-spoke model, northern pakistan, rural health, teleophthalmology

## Abstract

The hub-and-spoke model in ophthalmology was implemented in Northern Pakistan in 2021 under the Aga Khan Health Service, Pakistan (AKHS-P), in collaboration with the Aga Khan University, Karachi, to expand access to comprehensive and specialized eye care across the geographically challenging and historically underserved regions of Gilgit-Baltistan and Chitral.

A central ophthalmology hub was established in Gilgit, equipped with advanced diagnostic, laser, and surgical ophthalmic facilities to provide comprehensive eye care locally. The hub was staffed by a fellowship-trained ophthalmologist and supported by the Aga Khan University Hospital in Karachi as a super-hub, providing subspecialty expertise and teleophthalmology support. Two sub-hubs were subsequently established in Upper and Lower Chitral and staffed by optometrists, while peripheral spokes were developed across remote communities of Gilgit-Baltistan and Chitral and operated by frontline healthcare workers, with periodic visits by optometrists and ophthalmic technicians.

Through this tiered network, patients receive care at the level closest to their communities, while those requiring advanced diagnostic assessment, specialist consultation, or surgical intervention are referred to the appropriate level within the network. The sub-hubs address primary and routine ophthalmic needs and facilitate referral, whereas the central hub provides advanced diagnostic, laser, and surgical services. The super-hub at Aga Khan University Hospital, Karachi, further strengthens the network through subspecialty expertise, teleophthalmology, training, and clinical support. Future development may incorporate artificial intelligence (AI)-assisted tele-ophthalmology, continued human-resource development, and strengthened subspecialty networks to support sustainable delivery of comprehensive eye care across geographically isolated and resource-constrained settings.

## Introduction

The Global Burden of Disease Study 2017 estimated that 21.78 million out of Pakistan’s total population of 207.7 million were affected by blindness and vision impairment, with an age-standardized prevalence of 15.38% [1]. Although the burden of vision loss in Pakistan decreased by 21% between 1990 and 2017, the overall ophthalmic care infrastructure remains suboptimal. Specialized hospitals are scarce, basic health units are insufficiently equipped, and access to trained eye care providers is unevenly distributed across Pakistan. According to a report by the International Council of Ophthalmologists, there are 1,860 ophthalmologists in Pakistan, representing an average of 10 ophthalmologists per million population [2]. While this meets the World Health Organization recommendation for a target of four ophthalmologists per million, it is essential to note that most of these ophthalmologists are concentrated in urban centers, and only a few are trained in extracapsular cataract extraction and intraocular lens surgery [3]. These challenges are more prevalent in geographically remote and underserved regions, where the lack of infrastructure often limits access to tertiary care facilities. Northern Pakistan, which primarily includes Gilgit-Baltistan and Khyber Pakhtunkhwa and is home to the world’s tallest mountain ranges, is one such region where a scarcity of quality healthcare facilities, physician and staff shortages, and difficult transportation act as a barrier to health service provision and uptake [4]. Unfortunately, data regarding the prevalence of visual impairment in Northern Pakistan remains limited. A survey assessing the knowledge and behavior of people towards eye care and the barriers in seeking refractive services in the Hunza district of Gilgit-Baltistan found that approximately 97% of respondents reported insufficient refractive services, including a lack of refractive setup, eye care personnel, and spectacle dispensing centers in the region [5]. Additionally, only one-third of the participants reported visiting an ophthalmologist or optometrist to undergo evaluation for impaired vision, with up to 50% citing unavailability of services, followed by a lack of awareness and cost as the main barriers in seeking eye care [5]. Khan et al. found that the cataract surgical rate in Gilgit-Baltistan is 2,215 surgeries per million, with only 14.3% performed using the phacoemulsification technique, compared to rates in developed regions, which reach as high as 80% [6].

Despite the overall availability of ophthalmologists at the national level, their distribution remains geographically uneven, with specialist eye care services predominantly concentrated in urban centers, leaving rural and geographically isolated populations with comparatively limited access to specialist eye care [3]. Despite efforts by government, charitable, and non-governmental organizations to expand eye care services, substantial gaps in access persist, particularly in rural and geographically isolated communities. Further efforts are therefore needed to bridge these gaps and develop more equitable models of care delivery that address the unique challenges faced by underserved populations. One such model is the hub-and-spoke approach, which involves a central facility providing advanced care, supported by satellite spokes offering basic services in more remote areas. This model facilitates access to specialized services, optimizes resource allocation, and encourages collaboration between primary care providers and specialists. In the eye care context, this approach can significantly improve service delivery, enhance early detection and treatment, and prevent vision loss in underserved regions.

Here, we describe the development, implementation, and organization of a hub-and-spoke eye care model established by Aga Khan Health Service, Pakistan (AKHS-P), in Northern Pakistan, with particular emphasis on its service-delivery structure, referral pathways, implementation challenges, and lessons learned.

## Technical report

Development and implementation of the hub-and-spoke model

The model represents the first state-of-the-art ophthalmology setup in the underserved and remote regions of Gilgit-Baltistan and Chitral, where access to healthcare remains limited by high altitudes, mountainous terrain, and inadequate infrastructure. Gilgit-Baltistan spans 72,971 km² and serves a population of approximately 1.7 million across its three divisions: Chilas, Gilgit, and Baltistan. The initiative provides a scalable approach to delivering specialist ophthalmic care to geographically isolated communities. These divisions are further subdivided into fourteen districts. 

Upper and Lower Chitral, located in Khyber Pakhtunkhwa and adjacent to Gilgit-Baltistan, together cover an area of approximately 14,850 square kilometers and have a combined population of approximately 0.46 million (Figure 1). 

**Figure 1:**
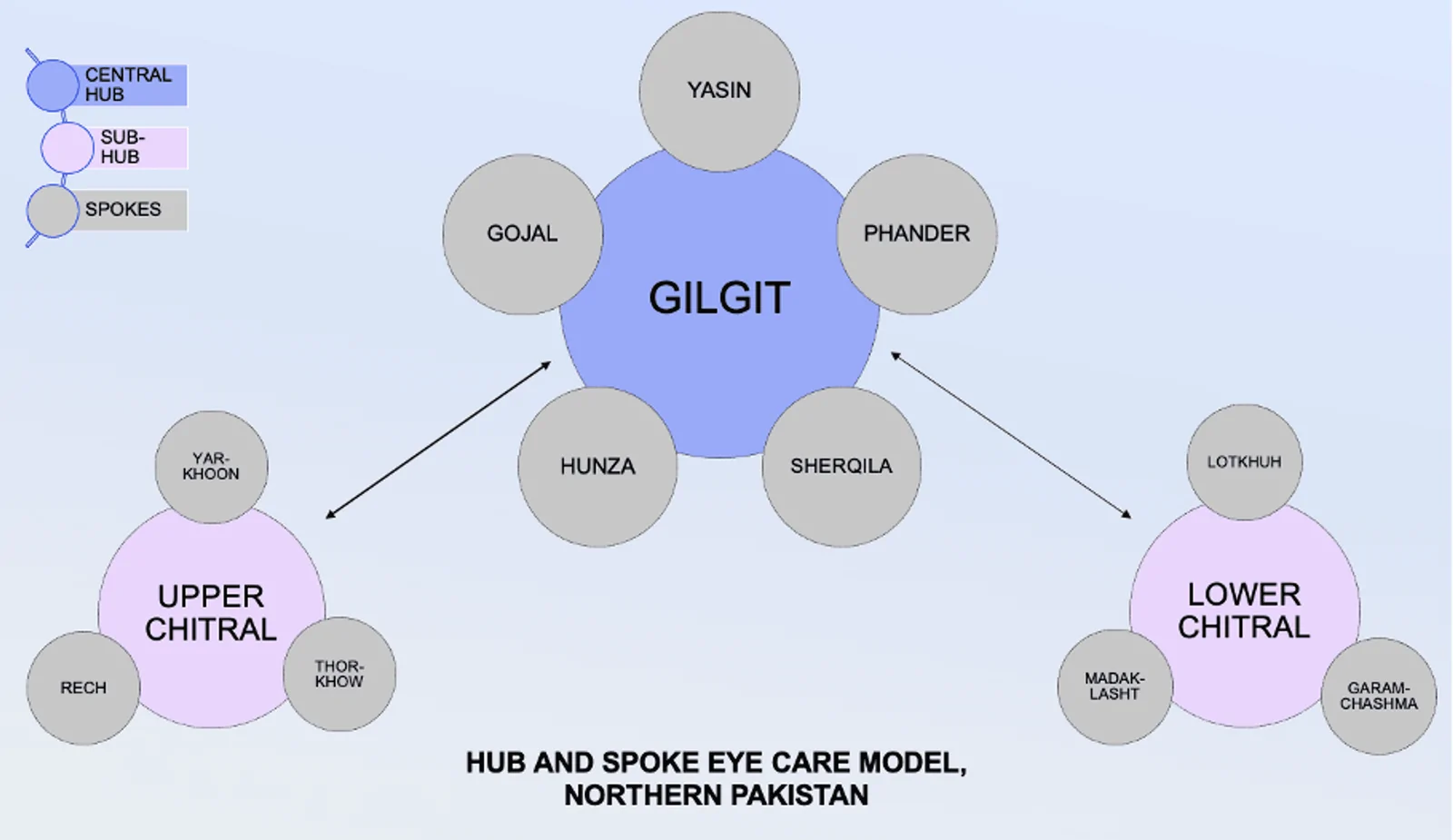
Hub-and-spoke eye care model in Northern Pakistan Image credits: Zubaida Sirang/Aga Khan Health Service, Pakistan; Created using Microsoft PowerPoint (Microsoft Corporation, Redmond, WA, USA).

The central hub was established in January 2021 in Gilgit, the administrative capital of the Gilgit-Baltistan region. This hub was staffed by a full-time fellowship-trained ophthalmologist, two part-time ophthalmologists, two optometrists, and six ophthalmic technicians. This was the first time that Aga Khan Hospital Gilgit was able to provide quality eye care that was accessible to individuals living in the central and surrounding regions. Previously, they were required to travel to larger urban centers to undergo advanced testing, including optical coherence tomography and perimetry, as well as treatments, including selective laser trabeculoplasty for open-angle glaucoma and pan-retinal photocoagulation for various retinal disorders. The central hub thus served as the anchor of our model, ensuring that a wide range of ophthalmic services, previously unavailable in the region, were now accessible to the local population without the need to travel long distances (Figure 2). 

**Figure 2:**
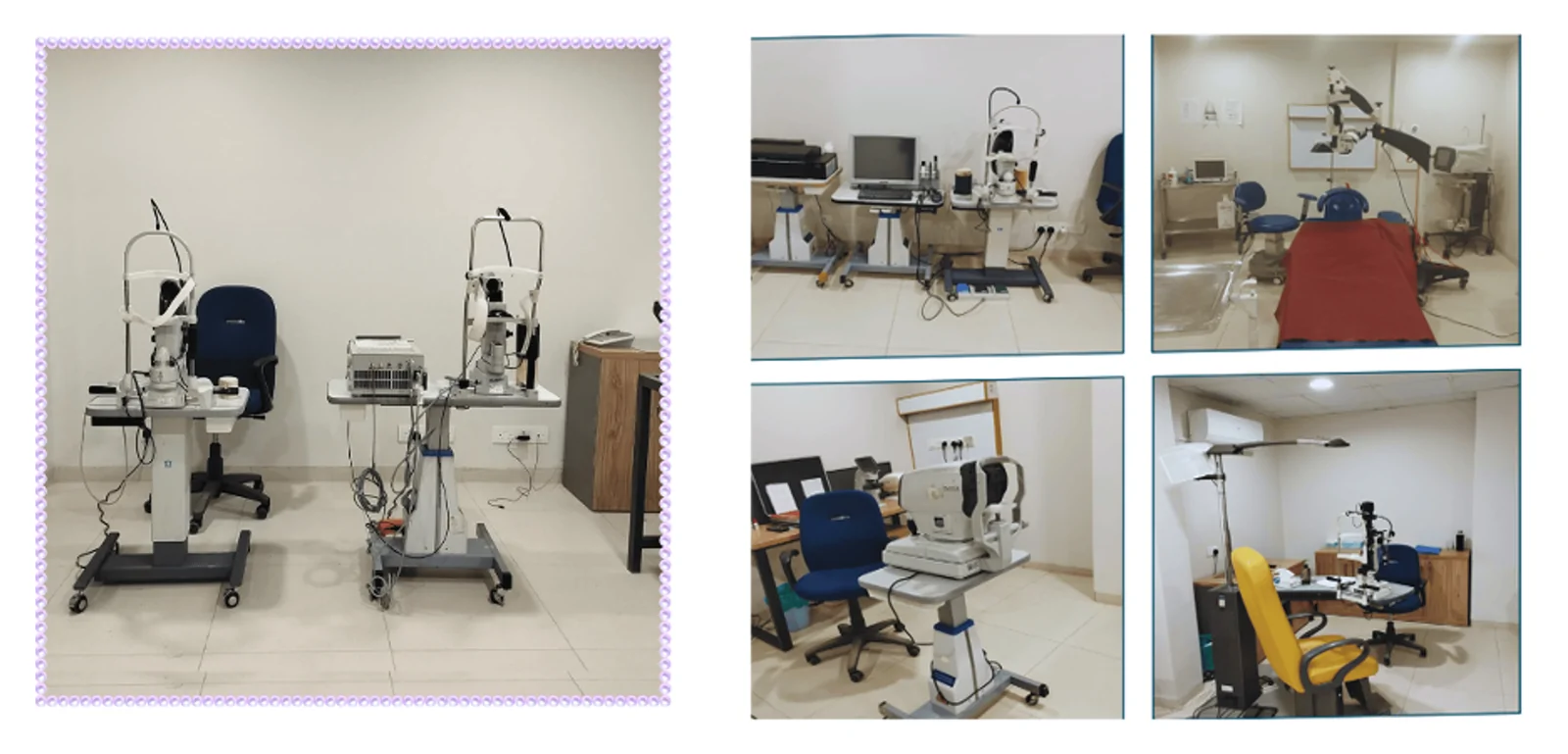
The central hub in Gilgit was equipped with advanced ophthalmic diagnostic and treatment facilities to support comprehensive eye care services. Image credits: Zubaida Sirang

The central hub was connected to the super-hub at Aga Khan University Hospital, Karachi, through teleophthalmology for subspecialty consultation and referral of patients requiring specialized care.

The sub-hubs, established in the districts of Upper and Lower Chitral, provided routine eye care and facilitated access to specialist and surgical services for surrounding communities. Each sub-hub was equipped with basic ophthalmic equipment and an operating room with a phacoemulsification machine. Optometrists were stationed full-time at the sub-hubs and served as the first point of contact, providing routine eye care, identifying patients who required specialist assessment, and coordinating referrals. Cataract and other ophthalmic surgeries were performed exclusively by visiting ophthalmologists during scheduled visits to the sub-hubs. Patients identified by optometrists as potential surgical candidates underwent ophthalmologist assessment and preoperative evaluation before surgery. Postoperative follow-up was coordinated between the visiting ophthalmologist and the resident optometrist, with referral or teleophthalmology consultation when further ophthalmologist assessment was required. Ophthalmologists generally visited the sub-hubs approximately every eight weeks for up to two weeks, during which they assessed complex cases, performed elective surgery, predominantly cataract surgery, and supported clinical training and capacity building.

Peripheral spokes were established in the rural areas of Gilgit-Baltistan and Chitral and were primarily staffed by Lady Health Visitors (LHVs). LHVs are frontline health workers employed by the AKHS-P, who provide primary care services to underserved populations in rural areas. They received basic ophthalmology training focused on eye screening and recognition of common eye conditions and ocular emergencies. They remained connected with optometrists and ophthalmologists through tele-ophthalmology for further assessment, guidance, and referral when required. Optometrists and ophthalmic technicians also visited the spokes routinely to conduct screening camps and deliver basic ophthalmic care. Patients with advanced eye conditions or those requiring surgery were then referred to sub-hubs or the central hub, depending on the level of care needed. As a result, the spokes became critical entry points within the care model, facilitating referrals and subsequent management while simultaneously providing care for common ocular problems (Figure 3).

**Figure 3:**
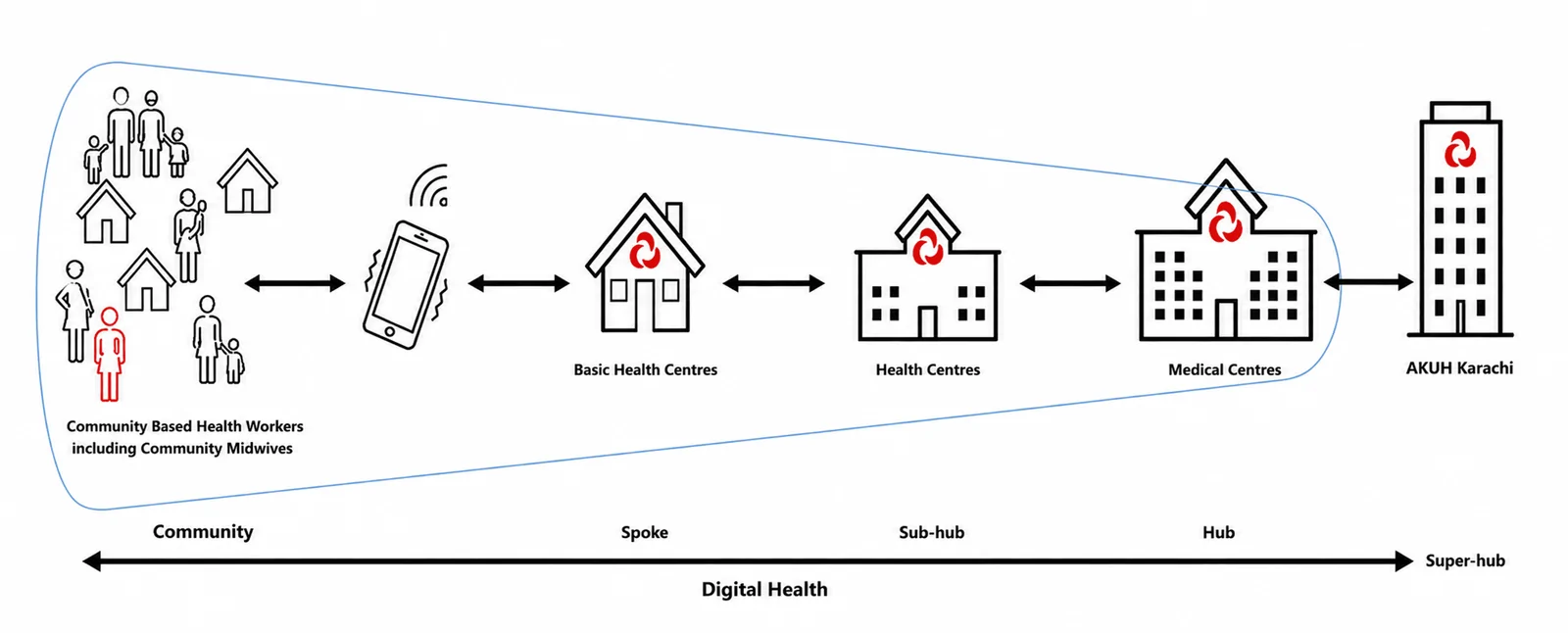
Hub-and-spoke eye care network connected to the super-hub at Aga Khan University Hospital (AKUH), Karachi This figure illustrates the integrated hub-and-spoke eye care model, linking community-based health workers and peripheral spokes with progressively higher levels of care at sub-hubs and the central hub. Digital health and tele-ophthalmology facilitate communication, consultation, and referral across the network. The central hub is further connected to the super-hub at AKUH, Karachi, for subspecialty consultation and referral of patients requiring specialized care. Image credits: Aga Khan Health Service, Pakistan. Created using Microsoft PowerPoint (Microsoft Corporation, Redmond, WA, USA).

Methodology and scope of service evaluation

This article is a descriptive technical report of a clinical service delivery model implemented from January 2021. The program was not designed as a prospective research study, and a standardized research database was not established during its initial implementation. Operational information for this report was obtained retrospectively from available AKHS-P program records covering January 2021 to December 2025, including outreach visit records and clinic and service records. Information was extracted by the lead author, who was directly involved in the implementation of the program, and cross-checked against available program records and clinical service documentation to verify the accuracy of the reported operational information. Because these records were collected for routine service delivery rather than research purposes, only information supported by the available program documentation was included. Comprehensive patient-level data with consistent denominators, year-by-year and site-by-site service volumes, referral completion rates, cataract surgical outcomes, postoperative visual acuity, and formal quality-of-life measures were not systematically collected in a format suitable for research analysis. Accordingly, this report focuses on the organization, staffing, referral pathways, services introduced, outreach activities, implementation challenges, and lessons learned rather than presenting unverified estimates as measured clinical outcomes. Prospective collection of standardized service indicators and clinical outcomes is planned as an important component of future evaluation. During the reporting period, ophthalmologists, optometrists, and ophthalmic technicians conducted 52 outreach visits to remote villages of Gilgit-Baltistan and Chitral. These outreach visits provided eye screening and opportunities to educate patients about the warning signs of serious eye conditions and ocular emergencies.

Patients requiring further assessment or management were directed to the sub-hubs or central hub according to clinical need. Cataract was among the conditions encountered through the network, and cataract surgery was provided as part of the surgical services. Refractive conditions managed through the network included refractive errors, complex prescriptions, pediatric refractive issues, and keratoconus. Patients with vernal keratoconjunctivitis and dry eye disease were also assessed and managed within the network.

The model also supported the assessment, management, and referral of patients with glaucoma and retinal disorders, including diabetic and hypertensive retinopathy and age-related macular degeneration. Corneal disorders, including infectious keratitis and corneal dystrophies, as well as eyelid and ocular surface disorders such as ptosis, entropion, ectropion, and blepharitis, were managed locally when appropriate or referred for specialist care according to clinical need.

Formal disease-specific data were not systematically collected. Therefore, the conditions described above are presented to illustrate the spectrum of ocular conditions encountered through the service and should not be interpreted as estimates of disease prevalence or relative frequency.

## Discussion

To the best of our knowledge, this is the first reported implementation of a hub-and-spoke model in Pakistan for delivering comprehensive eye care services. Our experience describes the establishment and continued operation of a coordinated, tiered eye care network in a geographically challenging and resource-constrained setting. By distributing basic services closer to communities while centralizing advanced diagnostic and surgical services, the model provides an organizational framework that may improve access and facilitate more efficient use of available resources and specialist expertise. However, these potential benefits were not formally evaluated in the present report.

Through the centralization of advanced diagnostic and surgical services at the central hub and the distribution of basic eye care services across sub-hubs and peripheral spokes, the model established a tiered network for delivering eye care across geographically challenging regions. Patients could receive care locally when appropriate and be referred to the central hub or a higher-level specialized center when required. By bringing selected ophthalmic services closer to patients’ communities, the model was designed to reduce the need for travel to distant urban centers and facilitate coordinated referral across different levels of care. However, travel requirements, patient expenditure, cost savings, visual outcomes, referral completion, and quality-of-life outcomes were not formally evaluated. Therefore, the clinical, economic, and patient-level impact of the model cannot be quantified from the present report. Prospective studies incorporating standardized service indicators and patient outcomes are required to evaluate these potential benefits.

The use of teleophthalmology by optometrists at sub-hubs and spokes supported remote assessment of patients and facilitated ophthalmologist input when in-person specialist assessment was difficult because of weather conditions, natural disasters, rugged terrain, or limited transportation infrastructure. Teleophthalmology has emerged as a promising approach for extending ophthalmic assessment and specialist input to underserved and resource-constrained settings. The e-Sanjeevni platform in India employed a hub-and-spoke model through teleophthalmology, serving over 5,000 patients during nine months, with 70% successfully diagnosed through teleconsultations [7]. Kassam et al. described a similar framework for delivering teleglaucoma at the University of Alberta, where information collected remotely by general ophthalmologists and optometrists at spoke sites was interpreted by a glaucoma specialist at the hub, who subsequently provided recommendations [8]. Shah et al. reported teleophthalmology as a cost-effective model for rural eye care in India, with an average saving of 621 INR per patient [9]. Although we did not perform a formal cost analysis, findings from other health systems provide contextual evidence regarding the potential economic benefits of teleophthalmology. This may be particularly relevant in Pakistan, where healthcare expenditure is primarily financed through out-of-pocket payments [10]. Similar economic benefits cannot, however, be assumed for the present model without formal evaluation.

Hub-and-spoke approaches have been reported across several areas of ophthalmic care, including cataract, refractive error, glaucoma, retinopathy of prematurity (ROP), microbial keratitis, and comprehensive eye care [8,11-16]. The Aravind Eye Hospital in South India has operated a hub-and-spoke system for several decades, combining community outreach with the training of mid-level ophthalmic personnel to support high-volume cataract services [11]. Nuzzi et al. proposed a pyramidal system for glaucoma care in Italy integrating public education, targeted screening, and early interventions [15]. Similarly, the Retina Eye Care of Nepal (RECON) project trained Master Eye Doctors through international partnerships, strengthened infrastructure at regional retina centers, developed human resources across ophthalmic cadres, and expanded community outreach through retina camps [16]. The Queen Elizabeth Diamond Jubilee Trust’s ROP initiative in India employed a modified hub-and-spoke model by developing screening and treatment capacity at medical college hubs that supported surrounding government Special Newborn Care Units as spokes, integrating ROP services into neonatal care across four states [13]. These examples demonstrate the application of tiered models across different ophthalmic settings and provide relevant context for the service delivery structure described in the present report.

Limitations, challenges, and future directions

A key limitation of this technical report is the absence of a prospectively designed service-evaluation database. Although routine clinical services were delivered throughout the network, patient-level data were not captured using uniform research definitions across all sites and years. Consequently, this report cannot provide validated denominators, annual or site-specific volumes, referral completion rates, standardized cataract surgical outcomes, postoperative visual acuity, quality-of-life outcomes, or economic outcomes. Approximate figures used during internal program planning were not considered sufficiently robust for formal outcome reporting and have therefore been excluded from the revised manuscript.

The most significant barrier we encountered while establishing our integrated eye care model was the lack of ophthalmologists and optometrists from the local community who had completed rigorous fellowship training and were familiar with the local language and culture. To address this shortage of human resources, a super-hub was established at the Aga Khan University Hospital, one of Pakistan's largest tertiary referral centers. The super-hub supported the network through teleophthalmology consultations, training and capacity building for optometrists and ophthalmic technicians at the sub-hubs and spokes, referral pathways for patients requiring subspecialty ophthalmic care, and opportunities for joint faculty appointments across participating institutions.

Although the distributed structure of the network was designed to bring services closer to remote communities, some satellite clinics remain distant from the central hub, and travel time was not formally evaluated in this report. These disparities are further exacerbated by poorly developed ground transportation, extreme weather conditions, and, particularly for women, safety concerns and cultural barriers that restrict independent travel. Periodic visits by ophthalmologists to sub-hubs, optometrists, and ophthalmic technicians to spokes have helped extend care to patients unable to access other clinics in the network. Yet, some gaps in coverage and equity persist.

Another limitation of our framework, particularly in tele-ophthalmology, is poor internet connectivity in northern Pakistan and its surrounding regions [17]. Although significant progress has been made in expanding high-speed internet infrastructure, especially following the COVID-19 pandemic, many rural areas are still deprived of even basic internet services, preventing the implementation of tele-ophthalmology setups in those areas. Re-envisioning future eye care would require a deliberate shift from fragmented service delivery toward a coordinated, equitable health system design that integrates human resource development, technological innovation, and interdisciplinary partnerships to address persistent disparities and extend care to the most vulnerable populations.

Collaborating with national and international eye hospitals and civil society organizations may further strengthen the current model by facilitating collaborative research and knowledge generation, sharing technical and human resources, and supporting capacity-building initiatives within local communities. By leveraging collective expertise, resources, and networks, these partnerships may promote sustainability, reduce the burden on individual institutions, and support culturally appropriate care through community engagement. Partnerships may also facilitate access to innovative financing mechanisms, including public-private partnerships, to support the expansion of specialized eye care in underserved regions. Such collaborations could further contribute to the development of regional centers of excellence and subspecialty training programs, including pediatric ophthalmology and vitreoretinal surgery fellowships, thereby strengthening the local workforce and supporting the region’s long-term eye care needs.

To further bridge existing gaps in access and extend services to communities in difficult and geographically isolated terrain, we are working toward incorporating a mobile eye clinic into the hub-and-spoke ophthalmology network. The mobile clinic could function as an extension of the existing spokes, bringing screening, basic diagnostic services, and referral pathways closer to communities that remain difficult to reach through fixed facilities. Patients requiring further assessment or treatment could subsequently be linked to the appropriate sub-hub, central hub, or super-hub according to the level of care required.

Furthermore, integrating artificial intelligence (AI) and machine learning (ML) into the teleophthalmology program may improve the efficiency of screening and identification of selected ocular conditions. Recent studies using AI and ML with fundus photography have demonstrated promising applications in the detection of diabetic retinopathy, glaucoma, age-related macular degeneration, and other ocular conditions [18-20]. Adopting similar approaches could complement the existing network, particularly in geographically isolated populations with limited access to specialist assessment. However, integrating these technologies requires careful local planning, sustainable resource allocation, appropriate imaging infrastructure, validation in the local population, and training of healthcare personnel. Future research should focus on long-term outcomes, cost-effectiveness, and sustained improvements in healthcare access associated with the integration of these technologies into hub-and-spoke systems.

## Conclusions

Our experience demonstrates the feasibility of implementing a community-based hub-and-spoke model to extend specialized eye care across geographically challenging and resource-constrained regions. By connecting peripheral communities with sub-hubs, a central ophthalmology hub, and subspecialty support through a super-hub, the model provides a structured framework for improving access to appropriate levels of ophthalmic care while making use of available local and specialist resources.

Sustaining and strengthening this model will require continued workforce development, quality assurance, appropriate use of technology, and collaboration across institutions. Prospective evaluation using standardized clinical, service, patient-reported, and economic indicators will be essential to determine its long-term effectiveness, sustainability, and applicability to other underserved settings.
